# Contemporary Clinical Spectrum and Factors Associated with Mortality in Infective Endocarditis: A Multicenter Cohort Study (COSMO-IE)

**DOI:** 10.3390/jcm15197760

**Published:** 2026-10-08

**Authors:** Mehmet Kış, Oktay Şenöz, Duygu İnan, Onur Akhan, Esra Canbolat Ünlü, Özlem Güler, Tuba Tatlı Kış, Şemsi Nur Karabela, Duygu Çerçioğlu Özdemir, Süleyman Çağan Efe, Pınar Şen, Hatice Özdamar, Derya Seyman, Ayşe Batırel, Utku Zeybey, Çisem Oktay, Pelin Geyik Pekin, Selin Özdemir, Adem Köse, Kemalettin Özden, Oğuz Karabay, Şafak Kaya Kavak, Ali Mert, Sena Kaçan, Tuncay Güzel, Selda Aydın, Uğur Kostakoğlu, Seyit Ali Büyüktuna, Mesut Gitmez, Esma Kepenek Kurt, Fatma Zühre Alparslan Tanyeri, Veysi Can, Lütfü Bekar, Mehmet Çelik, Nazan Tuna, Şükran Köse, Mehmet Birhan Yılmaz

**Affiliations:** 1 Department of Cardiology, Faculty of Medicine, Dokuz Eylul University, Izmir 35340, Türkiye; cisem.oktay@deu.edu.tr (Ç.O.); birhan.yilmaz@deu.edu.tr (M.B.Y.); 2 Department of Cardiology, University of Bakırçay, Izmir 35665, Türkiye; oktay.senoz@bakircay.edu.tr; 3 Department of Cardiology, Başakşehir Çam and Sakura City Hospital, Istanbul 34480, Türkiye; dr.duyguinan@gmail.com; 4 Department of Cardiology, Şehit Prof. Dr. İlhan Varank Sancaktepe Training and Research Hospital, Istanbul 34785, Türkiye; akhanonur@gmail.com; 5 Department of Infectious Diseases and Clinical Microbiology, University of Health Sciences, Bakırköy Dr. Sadi Konuk Training and Research Hospital, Istanbul 34147, Türkiye; dresracanbolatunlu@gmail.com (E.C.Ü.); semsinur.karabela@sbu.edu.tr (Ş.N.K.); 6 Department of Infectious Diseases and Clinical Microbiology, Faculty of Medicine, Kocaeli University, Kocaeli 41380, Türkiye; ozlemozkanguler@gmail.com; 7 Department of Infectious Diseases and Clinical Microbiology, University of Health Sciences, Dr. Suat Seren Chest Diseases and Surgery Training and Research Hospital, Izmir 35110, Türkiye; tubatatli@hotmail.com; 8 Department of Infectious Diseases and Clinical Microbiology, Kayseri City Hospital, Kayseri 38080, Türkiye; duygucercioglu@hotmail.com; 9 Department of Cardiology, Kartal Koşuyolu Training and Research Hospital, Istanbul 34865, Türkiye; scaganefe@gmail.com; 10 Department of Cardiology, Izmir Katip Çelebi University Atatürk Training and Research Hospital, Izmir 35360, Türkiye; pinarozdemirsen@gmail.com (P.Ş.); drselinozdemir@gmail.com (S.Ö.); 11 Department of Cardiology, Ankara Training and Research Hospital, Ankara 06230, Türkiye; haticeeozdamar@gmail.com; 12 Department of Infectious Diseases and Clinical Microbiology, University of Health Sciences, Antalya Training and Research Hospital, Antalya 07100, Türkiye; seymander@gmail.com; 13 Department of Infectious Diseases and Clinical Microbiology, University of Health Sciences, Istanbul Kartal Dr. Lütfi Kırdar City Hospital, Istanbul 34865, Türkiye; aysebatirel@yahoo.com (A.B.); plngeyik@gmail.com (P.G.P.); 14 Department of Cardiology, Tepecik Training and Research Hospital, Izmir 35020, Türkiye; utkuz1907@hotmail.com; 15 Department of Infectious Diseases and Clinical Microbiology, Faculty of Medicine, İnönü University, Malatya 44280, Türkiye; adem.kose@inonu.edu.tr (A.K.); sena.kacan@inonu.edu.tr (S.K.); 16 Department of Infectious Diseases and Clinical Microbiology, Faculty of Medicine, Atatürk University, Erzurum 25240, Türkiye; ozdenkemal@gmail.com; 17 Department of Infectious Diseases and Clinical Microbiology, Faculty of Medicine, Sakarya University, Sakarya 54187, Türkiye; drkarabay@yahoo.com; 18 Department of Infectious Diseases and Clinical Microbiology, University of Health Sciences, Gazi Yaşargil Training and Research Hospital, Diyarbakır 21070, Türkiye; ksafak76@gmail.com; 19 Department of Infectious Diseases and Clinical Microbiology, Istanbul Medipol University, Istanbul 34214, Türkiye; alimert@medipol.edu.tr (A.M.); seldaaydin@medipol.edu.tr (S.A.); 20 Department of Cardiology, University of Health Sciences, Gazi Yaşargil Training and Research Hospital, Diyarbakır 21070, Türkiye; drtuncayguzel@gmail.com; 21 Department of Infectious Diseases and Clinical Microbiology, Faculty of Medicine, Recep Tayyip Erdoğan University, Rize 53100, Türkiye; ugur.kostakoglu@erdogan.edu.tr (U.K.); zuhrealp7.3@gmail.com (F.Z.A.T.); 22 Department of Infectious Diseases and Clinical Microbiology, Faculty of Medicine, Sivas Cumhuriyet University, Sivas 58140, Türkiye; alibuyuktuna@gmail.com; 23 Department of Cardiology, Batman Training and Research Hospital, Batman 72070, Türkiye; drmesutgitmez@gmail.com; 24 Department of Infectious Diseases and Clinical Microbiology, Necmettin Erbakan University, Konya 42090, Türkiye; esma_kepenek@hotmail.com; 25 Department of Cardiology, Van Training and Research Hospital, Van 65170, Türkiye; veysican_droda72@hotmail.com; 26 Department of Cardiology, Faculty of Medicine, Hitit University, Çorum 19040, Türkiye; drlutfubekar@gmail.com; 27 Department of Infectious Diseases and Clinical Microbiology, Faculty of Medicine, Harran University, Şanlıurfa 63300, Türkiye; dr.mcelik12@gmail.com; 28 Department of Infectious Diseases and Clinical Microbiology, Faculty of Medicine, Tekirdağ Namık Kemal University, Tekirdağ 59030, Türkiye; tunanazan@hotmail.com; 29 Department of Infectious Diseases and Clinical Microbiology, Faculty of Medicine, Dokuz Eylul University, Izmir 35340, Türkiye; sukran.kose@deu.edu.tr

**Keywords:** infective endocarditis, hospital-acquired infection, *Staphylococcus aureus*, clinical complications

## Abstract

**Background**: Despite continuous advancements in diagnostic imaging and targeted antimicrobial protocols for infective endocarditis (IE), global epidemiological data show that both disease burden and incidence continue to rise. This study aimed to evaluate real-world diagnostic and treatment approaches in accordance with the 2023 European Society of Cardiology (ESC) IE Guidelines and to identify independent predictors of mortality in a contemporary patient cohort. **Methods**: This multicenter, retrospective cohort study analyzed data from 470 consecutive adult patients diagnosed with IE across 27 academic reference centers between 1 September 2023 and 1 June 2025. Patients were managed and adjudicated according to the 2023 ESC diagnostic criteria. Demographic data, clinical characteristics, microbiological profiles, imaging findings, empirical antibiotic therapy patterns, and clinical outcomes were analyzed. Independent predictors of mortality were determined using multivariable Cox proportional hazards regression models. **Results**: The mean age of the overall study population was 58.08 ± 16.81 years, with a male predominance (60.6%, *n* = 285). *Staphylococcus aureus* was the most frequently isolated pathogen (27.9%), with a high proportion of methicillin resistance (38.9%). Hospital-acquired IE was identified in 41.1% of cases. Surgical treatment was performed in 40.6% (*n* = 191) of the cohort. The 28-day mortality rate was 11.9%, and the overall mortality during follow-up reached 26.4%. Multivariable Cox regression analysis identified hospital-acquired IE (HR = 2.247; 95% CI: 1.216–4.167; *p* = 0.010) and the presence of major clinical complications (HR = 4.207; 95% CI: 1.556–11.378; *p* = 0.005) as the strongest independent predictors of mortality. **Conclusions**: Contemporary infective endocarditis is a highly complex and fatal disease characterized by a prominent healthcare-associated burden and a predominant staphylococcal etiology. The occurrence of major clinical complications and hospital-acquired infections were the primary independent predictors of mortality.

## 1. Introduction

Infective endocarditis (IE), situated at the intersection of cardiovascular medicine, infectious diseases, and clinical microbiology, remains a critical clinical condition characterized by varying clinical profiles, an altered microbiological spectrum, and persistently high mortality rates [1,2]. Despite continuous advancements in diagnostic imaging and targeted antimicrobial protocols, global epidemiological data show that both disease burden and incidence continue to rise [2,3]. Over the past few decades, the predisposing factors for IE have undergone a significant transformation; while the proportion of classical substrates such as rheumatic valvular disease has declined, there has been a marked increase in infections associated with degenerative valvular disease, prosthetic valve replacement, and cardiac implantable electronic devices (CIEDs) [1,4].

IE occurs with a higher incidence in males, elderly individuals, and patients with chronic kidney disease (particularly those undergoing hemodialysis), as well as in those who have undergone solid organ or hematopoietic stem cell transplantation, an intravascular catheter, or predisposing cardiac conditions compared to the general population. Cardiac conditions predisposing to IE include the presence of intracardiac foreign bodies such as prosthetic valves, cardiac implantable electronic devices, or ventricular assist devices, as well as a history of prior IE, structural valvular heart diseases, and congenital heart diseases [1,5,6]. Streptococci or staphylococci are the causative agents in 80% of IE cases. In recent years, as a result of the increase in healthcare-associated IE cases, *S. aureus* and coagulase-negative staphylococci have become more frequent pathogens, whereas the proportion of viridans group streptococci has relatively decreased [6,7,8].

Improving clinical outcomes in IE requires rapid diagnostic strategies and a structured multidisciplinary approach [5]. Identifying the causative microorganism in IE is critical for diagnosis and guiding antimicrobial treatment. Mortality increases when the causative agent of IE cannot be identified [1,4,5]. While transthoracic (TTE) and transesophageal echocardiography (TEE) remain the primary diagnostic methods, cardiac computed tomography (CT), magnetic resonance imaging (MRI), and nuclear imaging techniques are also used to evaluate paravalvular complications, prosthetic valve dysfunction, and embolic foci. Effective antimicrobial treatment in IE is crucial for survival. Timely and appropriate selection of antibiotics in the management of IE reduces the development of severe sepsis, multiple organ failure, the risk of embolism, morbidity, and mortality [7].

The 2023 European Society of Cardiology (ESC) Guidelines introduced significant innovations regarding the updating of diagnostic criteria, the integration of novel imaging modalities into the system, and the clarification of early surgical indications in high-embolic-risk cases [1]. However, more data are needed on adopting these guideline recommendations in real-world clinical practice. In recent years, healthcare-associated infective endocarditis has emerged as a particularly vulnerable and high-risk clinical phenotype, driven by the increasing burden of invasive medical procedures, chronic hemodialysis, and frequent healthcare encounters. Unlike traditional community-acquired cases, healthcare-associated endocarditis is frequently associated with resistant pathogens, delays in diagnosis and treatment, and a much heavier burden of comorbidities; all of these factors contribute to an unacceptably high mortality rate. Despite its growing epidemiological importance, real-world data regarding the distinct prognostic impact and clinical course of healthcare-associated endocarditis remain limited [8,9]. In particular, the impact of current microbiological shifts on clinical outcomes and the independent predictors of survival in complex patient cohorts remain relevant research topics [4,6].

This study aims to evaluate real-world diagnostic and treatment approaches following the current ESC IE guideline. Subsequent aims are to examine the demographic characteristics, underlying microbiological agents, and anatomical complications of IE patients, as well as to identify independent clinical, echocardiographic, and biochemical parameters associated with mortality. Specifically, to address the gaps in real-world clinical practice, this multicenter study focuses on three primary objectives: (1) evaluating whether clinical centers adhere to the 2023 ESC recommendations for empirical antibiotic therapy in light of the changing spectrum of responsible bacteria and determining the impact of appropriateness on prognosis; (2) assessing the precise prognostic impact and clinical implications of hospital-acquired infective endocarditis; and (3) refining the independent in-hospital determinants and predictors of survival in a contemporary nationwide cohort of definite endocarditis patients following this guideline.

## 2. Materials and Methods

### 2.1. Study Design and Setting

This multicenter, retrospective cohort study was conducted across 27 academic reference centers, utilizing pooled data from specialized clinical divisions of cardiology, infectious diseases, and clinical microbiology. Patient demographics, clinical characteristics, and laboratory variables were systematically extracted from integrated electronic medical archives and registry databases for consecutive adult patients managed between 1 September 2023 and 1 June 2025. The study protocol was conducted in strict compliance with the ethical commitments of the Declaration of Helsinki. Administrative approval for data collection and analysis was granted by the Medical Ethics Committee of the coordinating academic institution.

### 2.2. Patient Selection and Diagnostic Definitions

All adult individuals (>18 years) admitted to the participating hospital with a diagnosis of IE during the observation period were screened for eligibility. To ensure methodological consistency and eliminate diagnostic bias across the study timeline, all historical and active cases were systematically re-evaluated and adjudicated based on the 2023 ESC Endocarditis Guidelines Diagnostic Criteria [1]. Only patients fulfilling the criteria for “Definite IE” as defined by the updated guidelines were included in the statistical analysis. To prevent statistical confounding linked to recurrent infectious episodes, only the index presentation of IE for each patient was considered.

The exclusion criteria were defined as

1. Patient age under 18 years;
2. A history of out-of-hospital cardiopulmonary resuscitation or a state of refractory cardiogenic shock at the time of initial emergency department evaluation;
3. Missing or substantially incomplete clinical, microbiological, or echocardiographic documentation within the registries.

### 2.3. Clinical, Microbiological, and Echocardiographic Evaluation

Baseline clinical profiles, including age, gender, detailed comorbidities (hypertension, diabetes mellitus, chronic kidney disease, and prior endocardial infections), and predisposing structural substrates (prosthetic heart valves, central venous catheters, or indwelling CIEDs) were thoroughly documented. Individual cases were classified by mode of acquisition into community-acquired IE or hospital-acquired IE. Specifically, hospital-acquired IE comprised infections developing at least 48 h after hospital admission or those diagnosed within 48 h in patients with significant recent healthcare exposures (such as maintenance hemodialysis, recent hospitalization, or chronic central venous catheterization), thereby ensuring that these clinical risk factors were appropriately accounted for within the acquisition classification.

Microbiological evaluation involved analyzing data from consecutive peripheral blood cultures, serological assays, and intraoperative valve tissue cultures or targeted molecular assays where available. Microbiological response was defined as the documentation of blood culture negativization evaluated on the 7th day of appropriate antimicrobial therapy in culture-positive patients. Complete echocardiographic examinations (comprising transthoracic and subsequent transesophageal evaluations as clinically indicated) performed by certified imaging specialists were comprehensively reviewed. Advanced anatomical destruction parameters were systematically recorded during the index hospitalization, specifically focusing on

- Maximum vegetation length (stratified at standard clinical cut-offs of >10 mm, and critical measurements > 15 mm);
- Development of perivalvular abscesses or pseudoaneurysms;
- New-onset or progressive paravalvular leakage (PVL);
- Valve leaflet disruption or perforation.

### 2.4. Protocol for Empirical Antimicrobial Therapy

Data regarding the administration of initial empirical antimicrobial regimens were systematically evaluated to assess adherence to contemporary international standards. Medical records were reviewed to verify whether a standardized protocol requiring three consecutive sets of blood cultures obtained at thirty-minute intervals had been consistently followed prior to initiating antimicrobial agents. The selection of initial empirical therapy was individualized based on several key clinical determinants:

Prior Treatment Status: Adjustments were applied for individuals with a recent history of antimicrobial therapy to ensure adequate and non-overlapping spectrum.

Infection Substrate and Valve Status: Therapeutic strategies for native valve infections and late prosthetic valve involvement were structured to target staphylococcal, streptococcal, and enterococcal pathogens, with coagulase-negative staphylococci specifically incorporated into prosthetic valve protocols. Conversely, early prosthetic or healthcare-associated episodes required an expanded antimicrobial spectrum encompassing methicillin-resistant staphylococci, enterococci, and non-HACEK Gram-negative bacteria.

Epidemiological Context and Acquisition Origin: Stratification between community-onset and healthcare-associated acquisition was evaluated alongside institutional resistance profiles.

Microbiological Customization: Following initial administration, regimens were swiftly adjusted to pathogen-directed targeted therapy once definitive susceptibility reports became available, typically within a 24-to-48-h window.

Appropriate empirical antimicrobial therapy was defined as the initiation of an antimicrobial regimen fully concordant with current international guidelines, taking into account native versus prosthetic valve status, healthcare-associated versus community-acquired acquisition, and institutional epidemiological resistance patterns, and subsequently optimized according to blood culture isolation and susceptibility results.

### 2.5. Endpoint Assessment and Clinical Stratification

The study cohort was stratified into two distinct clinical groups based on the primary clinical outcome documented during the follow-up period:

- Group 1 (Mortality): Patients who died during the follow-up period.
- Group 2 (Survivors): Patients who completed their prescribed medical and/or surgical therapy and were alive at the end of available follow-up.

Secondary clinical complications monitored across the retrospective observation window included the incidence of acute congestive heart failure, systemic or peripheral embolic events (including central nervous system embolic stroke, visceral splenic infarction, and acute pulmonary embolism), persistent uncontrolled bacteremia, new-onset cardiac arrhythmias, and acute myocardial infarction (AMI). Additionally, adherence to guideline-directed medical therapy (GDMT) and the formal integration of a collaborative Endocarditis Team pathway (joint management involving cardiology, infectious disease, and cardiothoracic surgery) were evaluated to determine their direct prognostic association with in-hospital survival.

### 2.6. Statistical Analysis

The distribution of continuous data was formally evaluated for normality using visual diagnostics (frequency histograms and normal Q–Q plots) and confirmed analytically using the Kolmogorov–Smirnov and Shapiro–Wilk tests. Normally distributed continuous variables were presented as mean ± standard deviation (SD), whereas non-normally distributed continuous variables were reported as medians with interquartile ranges (IQR). Categorical variables were presented as absolute frequencies and percentages.

Inter-group differences in baseline characteristics, microbiological patterns, and echocardiographic findings between survivors and non-survivors were analyzed using the independent Student’s *t*-test or the Mann–Whitney U test for continuous variables and the chi-square or Fisher’s exact test for categorical variables, depending on the cell distribution. Survival probabilities were estimated using the Kaplan–Meier method, and survival distributions were compared using the log-rank test. Univariable Cox proportional hazards regression analyses were performed to identify factors associated with all-cause mortality during follow-up. Clinically relevant variables and those with a univariable *p*-value < 0.10 were considered for inclusion in a multivariable Cox regression model. Adjusted hazard ratios with 95% confidence intervals were reported. The proportional hazards assumption was assessed using Schoenfeld residuals. To evaluate the impact of disease progression during follow-up, parameters demonstrating significant differences between survivors and non-survivors were incorporated into a composite clinical complications endpoint for both univariable and multivariable regression analyses. Statistical significance was defined as a two-sided *p*-value < 0.05. Statistical analyses were conducted using IBM SPSS Statistics (version 32.0; IBM Corp., Armonk, NY, USA) and R (version 4.6.0; R Foundation for Statistical Computing, Vienna, Austria).

## 3. Results

### 3.1. Baseline Characteristics and Clinical Presentation

A total of 470 consecutive adult patients diagnosed with infective endocarditis (IE) were evaluated across the participating tertiary academic centers. The mean age of the overall study population was 58.08 ± 16.81 years, with a male predominance (60.6%, *n* = 285). Regarding baseline comorbidities, hypertension was the most frequent condition (56.8%, *n* = 267), followed by diabetes mellitus (35.7%, *n* = 168), chronic kidney disease (34.5%, *n* = 162), and coronary artery disease (32.6%, *n* = 153). Furthermore, a history of stroke or transient ischemic attack was documented in 20.4% (*n* = 96) of the cohort, while congestive heart failure was present in 26.4% (*n* = 124) (Table 1). When analyzing clinical presentation, fever was the most common symptom, observed in 61.5% (*n* = 289) of patients, followed by dyspnea in 44.9% (*n* = 211), myalgia in 26.2% (*n* = 123), and arthralgia in 21.9% (*n* = 103).

### 3.2. Acquisition Settings, Anatomical Distribution, and Predisposing Conditions

Native valve endocarditis accounted for the majority of cases (65.7%, *n* = 309), whereas prosthetic valve endocarditis and cardiac implantable electronic device (CIED)-related endocarditis were identified in 24.5% (*n* = 115) and 6.6% (*n* = 31) of patients, respectively. In terms of acquisition setting, 58.9% (*n* = 277) of infections were community-acquired, and 41.1% (*n* = 193) were hospital-acquired. Anatomically, the mitral valve was the most frequently affected site (48.1%, *n* = 226), followed by the aortic valve (30.4%, *n* = 143) and the tricuspid valve (11.7%, *n* = 55). Prominent predisposing conditions and healthcare exposures included underlying valvular heart disease (50.6%, *n* = 238), recent hospitalization within the previous 6 months (45.2%, *n* = 212), prosthetic valves (26.0%, n = 122), central venous catheters (25.5%, *n* = 120), and chronic hemodialysis (24.5%, *n* = 115) (Table 2).

### 3.3. Microbiological Profile

Microbiological evaluation revealed *Staphylococcus aureus* as the most prevalent causative pathogen, identified in 27.9% (*n* = 131) of patients, followed by *Enterococcus* spp. (13.4%, *n* = 63), coagulase-negative staphylococci (12.1%, *n* = 57), and *Streptococcus* spp. (7.9%, *n* = 37). Culture-negative infective endocarditis was recorded in 15.7% (*n* = 74) of the cohort. Among staphylococcal isolates with available susceptibility testing, methicillin-resistant strains accounted for 45.3% (*n* = 105) (Table 3).

### 3.4. Clinical Presentation and Echocardiographic Findings

At presentation, fever was the most common symptom, observed in 61.5% (*n* = 289) of patients, followed by dyspnea in 44.9% (*n* = 211), myalgia in 26.2% (*n* = 123), and arthralgia in 21.9% (*n* = 103). Echocardiographic evaluation demonstrated mobile vegetations in 59.6% (*n* = 280) of patients and moderate-to-severe valvular regurgitation in 51.1% (*n* = 240). Additional structural complications included paravalvular leaks in 20.0% (*n* = 94), flail leaflets in 12.1% (*n* = 57), prosthetic valve dehiscence in 8.1% (*n* = 38), and paravalvular abscesses in 6.4% (*n* = 30) (Table 4). Empirical antimicrobial therapy was administered to 89.8% (*n* = 422) of the study population, with vancomycin (45.7%, *n* = 215) and third-generation cephalosporins (36.9%, *n* = 173) serving as the most frequently prescribed initial agents. Among patients who received empirical antimicrobial therapy, treatment was classified as appropriate in 216 of 422 patients (51.2%), inappropriate in 170 (40.3%), and not assessable in 36 (8.5%) (Table 4).

### 3.5. Clinical Complications, Treatment Modifications, and Outcomes

During the index hospitalization, various systemic complications developed, most notably new-onset or worsening heart failure (27.9%, *n* = 131/470), septic embolism (25.1%, *n* = 118/470), sepsis (20.9%, *n* = 98/470), cerebral embolism (18.9%, *n* = 89/470), acute kidney injury (13.4%, *n* = 63/470), and cardiac arrhythmias (12.3%, *n* = 58/470). Support measures included endotracheal intubation in 16.8% (*n* = 79) and total parenteral nutrition in 7.0% (*n* = 33) of patients (Table 5). Treatment regimens were subsequently modified based on culture results in 49.9% (*n* = 224/449) of patients, due to inadequate clinical response in 15.9% (*n* = 73/460), and due to adverse drug effects in 9.8% (*n* = 45/459). Overall, a favorable clinical response by day 7 was achieved in 67.2% (*n* = 311/463) of patients, and microbiological response was documented in 65.7% (*n* = 290/396). Surgical treatment decisions were made for 40.6% (*n* = 191) of the cohort, while outpatient management was selected for 25.3% (*n* = 119). The 28-day mortality rate was 11.9% (*n* = 56), and the mortality during follow-up was 26.4% (*n* = 124) (Table 5).

### 3.6. Comparative Analysis Between Survivors and Non-Survivors

Comparative evaluations between survivors (*n* = 346) and non-survivors (*n* = 124) demonstrated several significant differences. The mean age was significantly higher among non-survivors than survivors (62.73 ± 17.17 vs. 56.42 ± 16.38 years, *p* < 0.001). Non-survivors had a higher prevalence of baseline heart failure (34.7% vs. 23.4%, *p* = 0.015), underlying valvular heart disease (60.5% vs. 47.1%, *p* = 0.011), and central venous catheter exposure (33.9% vs. 22.5%, *p* = 0.013). Furthermore, hospital-acquired acquisition settings were significantly more common among non-survivors compared to survivors (59.7% vs. 34.4%, *p* < 0.001) (Table 6).

**Figure 1:**
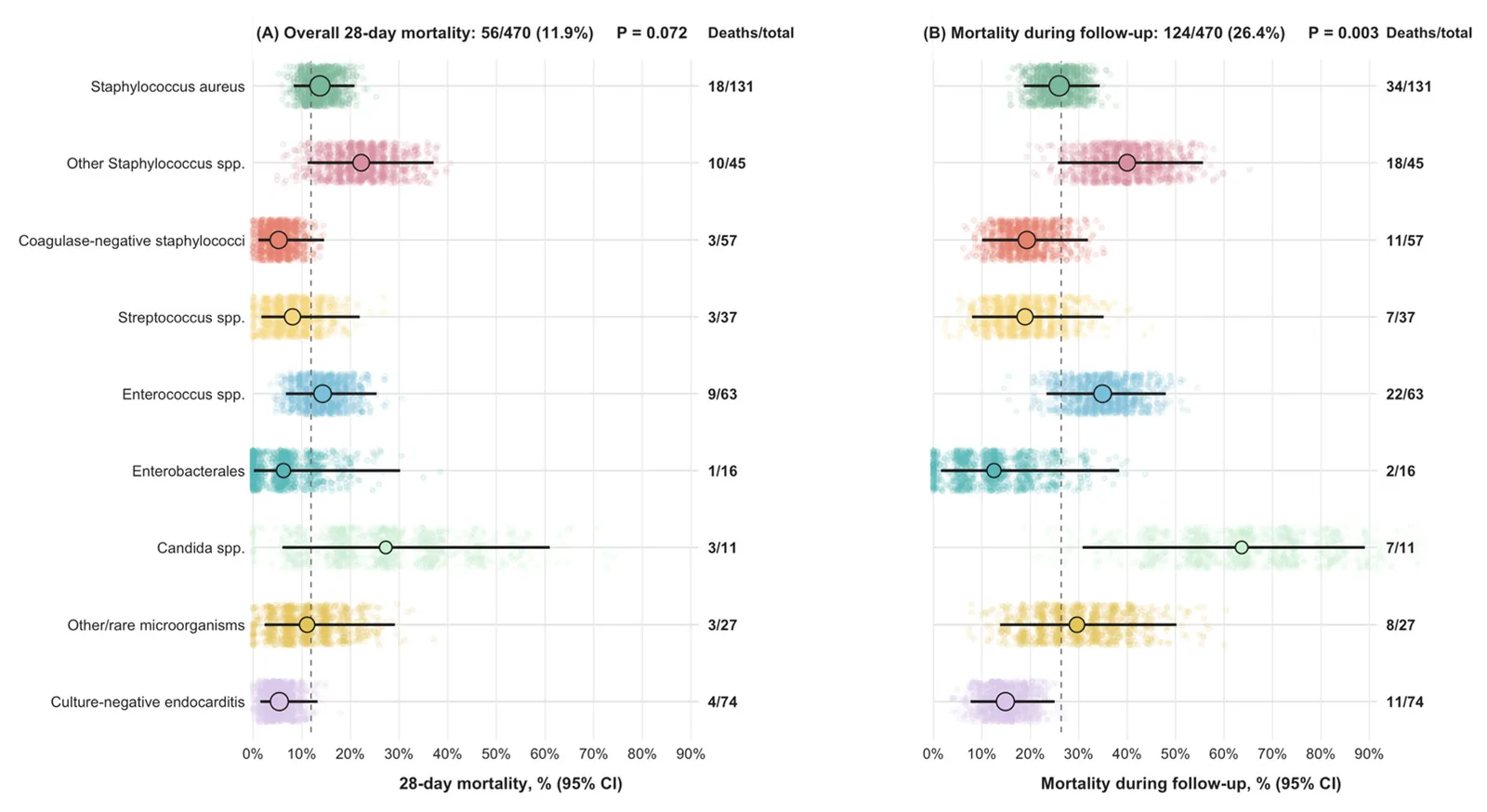
Mortality outcomes according to microbiological aetiology. (**A**) Overall 28-day mortality stratified by microbiological aetiology. (**B**) Mortality during follow-up stratified by microbiological aetiology. Point estimates represent observed mortality proportions, horizontal bars denote exact 95% confidence intervals, and dashed vertical lines indicate the overall mortality rate within each panel. For graphical presentation, non-fermentative Gram-negative bacilli, *Brucella* spp., and other uncommon pathogens were combined into the “Other/rare microorganisms” category. Nine patients with polymicrobial infection or unclassified microbiological findings were not included in the figure. Note: Different colors correspond to the distinct microorganism groups and species displayed on the vertical axis.

Mortality differed according to microbiological aetiology during follow-up (*p* = 0.003), whereas the between-group difference in 28-day mortality did not reach statistical significance (*p* = 0.072). Candida spp. was associated with the highest observed mortality estimates, whereas culture-negative endocarditis showed comparatively lower estimates (Figure 1).

Non-survivors more frequently presented with dyspnea (53.2% vs. 41.9%, *p* = 0.030). Echocardiographically, non-survivors exhibited a significantly higher frequency of mobile vegetations (76.6% vs. 53.5%, *p* < 0.001) and moderate-to-severe valvular regurgitation (64.5% vs. 46.2%, *p* < 0.001) (Table 7). The non-survivors group had significantly longer post-endocarditis intensive care unit stays than survivors (12.0 [5.0–24.0] vs. 7.0 [3.0–13.0] days, *p* = 0.002). Post-discharge follow-up duration is presented descriptively and was not considered a prognostic variable (Table 8).

Analysis of laboratory parameters revealed non-survivors had higher serum creatinine levels (1.60 [0.99–4.15] vs. 1.19 [0.83–2.89] mg/dL, *p* = 0.013) and CRP concentrations (109.0 [48.0–207.3] vs. 78.0 [14.0–153.0] mg/L, *p* = 0.001), together with lower eGFR values (41.0 [12.5–71.0] vs. 63.0 [22.0–91.0] mL/min/1.73 m^2^, *p* < 0.001). Non-survivors had significantly lower hemoglobin levels (10.02 ± 2.12 vs. 10.77 ± 2.43 g/dL, *p* = 0.002) and serum albumin (3.09 ± 0.87 vs. 3.32 ± 1.00 g/dL, *p* = 0.034), alongside higher white blood cell counts (13.24 ± 6.50 vs. 11.72 ± 5.63 x10^9^/L, *p* = 0.022) and systolic pulmonary artery pressures (38.34 ± 15.63 vs. 34.59 ± 13.57 mmHg, *p* = 0.025) (Table 8).

Regarding empirical antimicrobial management, non-survivors presented a numerically higher rate of empirical antibiotic administration compared to survivors (94.4% vs. 88.2%, *p* = 0.050). While initial treatment modifications based on culture results were similar between groups, non-survivors required significantly more frequent regimen modifications due to a lack of clinical response (25.8% vs. 12.4%, *p* = 0.001). Consequently, non-survivors demonstrated markedly lower rates of favorable clinical response at day 7 (45.9% vs. 74.8%, *p* < 0.001) and microbiological clearance (52.9% vs. 66.1%, *p* = 0.010) (Table 9).

Clinical complications were substantially more frequent among non-survivors, including sepsis (46.0% vs. 11.8%, *p* < 0.001), septic embolism (52.4% vs. 15.3%, *p* < 0.001), cerebral embolism (37.9% vs. 12.1%, *p* < 0.001), acute kidney injury (34.7% vs. 5.8%, *p* < 0.001), new-onset or worsening heart failure (37.9% vs. 24.3%, *p* = 0.004), arrhythmias (24.2% vs. 8.1%, *p* < 0.001), and myocardial infarction (12.1% vs. 3.2%, *p* < 0.001). Other complications, including spondylodiscitis, immune complex nephritis, splenic abscess, and pulmonary embolism, were also significantly more frequent among non-survivors (all *p* < 0.05) (Table 9).

Univariable and multivariable Cox proportional hazards regression analyses were performed to identify independent predictors of mortality. In the univariable analysis, several variables including age (per 1-year increase: HR = 1.027, 95% CI: 1.011–1.043, *p* = 0.001), hospital-acquired infective endocarditis (HR = 2.101, 95% CI: 1.312–3.367, *p* = 0.002), mobile vegetation (HR = 2.005, 95% CI: 1.161–3.461, *p* = 0.013), eGFR (HR = 0.989, 95% CI: 0.983–0.995, *p* = 0.001), hemoglobin (HR = 0.842, 95% CI: 0.747–0.950, *p* = 0.005), systolic pulmonary artery pressure (HR = 1.019, 95% CI: 1.004–1.035, *p* = 0.015), ICU length of stay (HR = 1.019, 95% CI: 1.010–1.028, *p* < 0.001), fosfomycin use (HR = 9.161, 95% CI: 2.193–38.269, *p* = 0.002), treatment modification due to lack of response (HR = 2.593, 95% CI: 1.535–4.381, *p* = 0.001), clinical response at day 7 (HR = 0.397, 95% CI: 0.247–0.637, *p* < 0.001), microbiological response (HR = 0.529, 95% CI: 0.328–0.853, *p* = 0.009), clinical complications (HR = 6.649, 95% CI: 3.175–13.925, *p* < 0.001), and outpatient treatment (HR = 0.476, 95% CI: 0.297–0.762, *p* = 0.002) demonstrated significant associations with mortality, whereas the administration of any empirical antibiotic therapy showed no significant association (HR = 1.522, 95% CI: 0.659–3.514, *p* = 0.325) (Table 10). However, in the multivariable Cox regression model, only hospital-acquired infective endocarditis (HR = 2.247, 95% CI: 1.216–4.167, *p* = 0.010) and the presence of clinical complications (HR = 4.207, 95% CI: 1.556–11.378, *p* = 0.005) emerged as independent predictors of mortality, whereas age showed a borderline association (HR = 1.021, 95% CI: 1.000–1.043, *p* = 0.053) (Table 10).

To address the contributing components of the composite clinical complications endpoint and evaluate the distinct prognostic contribution of individual events, univariable and multivariable Cox proportional hazards regression analyses were performed for each specific complication (Supplementary Table S1). While multiple individual events demonstrated significant prognostic value in univariable models, sepsis, acute kidney injury, cranial embolism, and myocardial infarction remained independent predictors in multivariable analyses.

**Figure 2:**
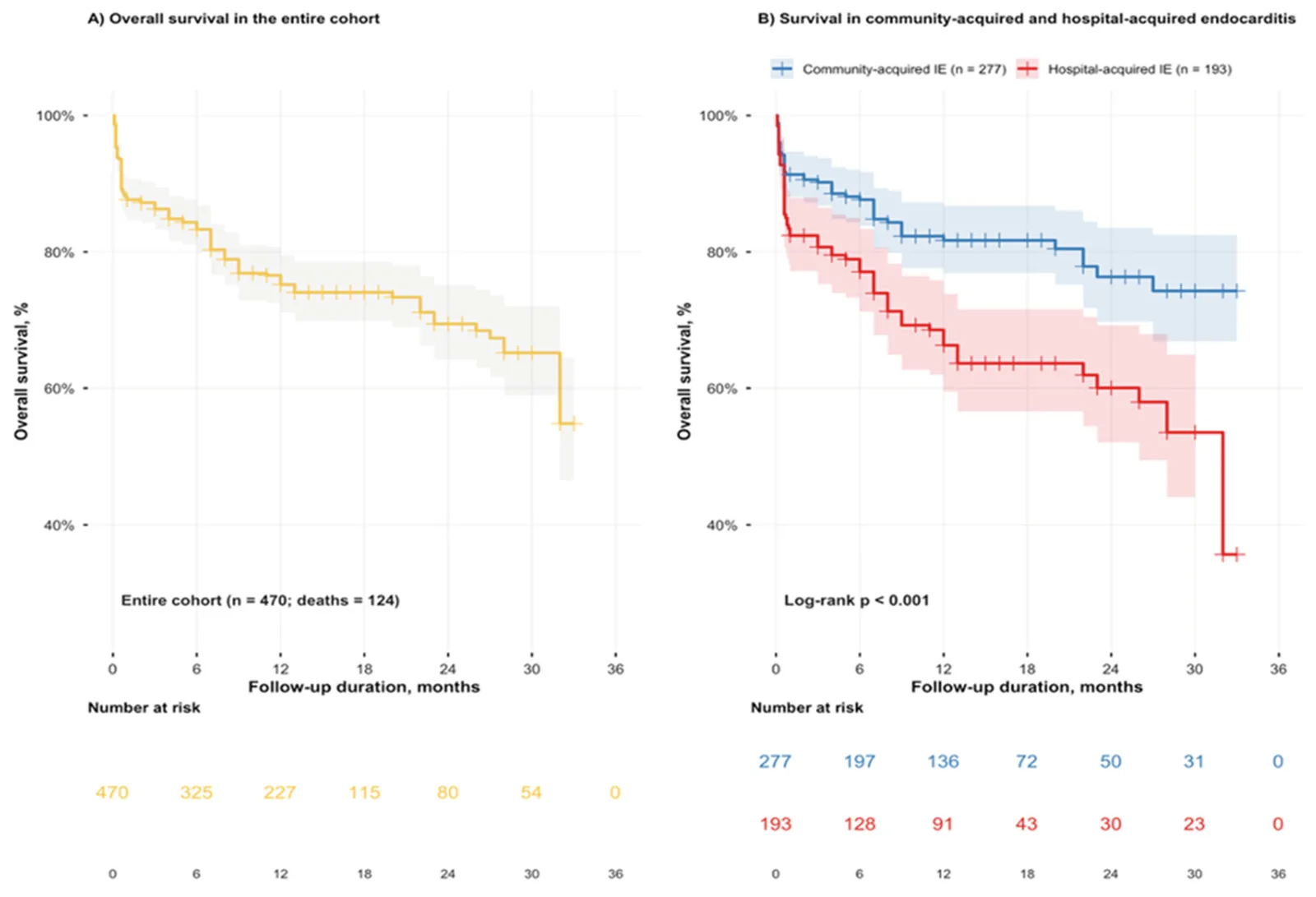
Kaplan–Meier survival curves for the overall cohort and for community-acquired and hospital-acquired endocarditis. (**A**) Overall survival in the entire cohort. (**B**) Survival in patients with community-acquired and hospital-acquired endocarditis. Shaded areas represent 95% confidence intervals, vertical marks indicate censored observations, and the numbers at risk are shown below each panel.

During follow-up, 124 of 470 patients died, corresponding to an overall mortality rate of 26.4%. Patients with hospital-acquired infective endocarditis had significantly poorer overall survival than those with community-acquired infection (log-rank *p* < 0.001) (Figure 2).

## 4. Discussion

This large, multicenter, retrospective cohort study involving 470 adult patients evaluated contemporary clinical profiles, microbiological spectrum, management strategies, and predictors of mortality in IE following the publication of the 2023 ESC Guideline. Our findings provide critical real-world insights into the evolving spectrum of IE. In accordance with our study objectives, the primary findings are structured as follows: (1) evaluating empirical antimicrobial therapy in light of the changing bacterial spectrum revealed that *Staphylococcus aureus* (27.9%), with a high rate of methicillin resistance (38.9%), has consolidated its position as the predominant causative pathogen, driven by a major epidemiological shift toward hospital-acquired infections (41.1%); (2) assessing the precise prognostic impact of clinical complications demonstrated that in-hospital adverse events, particularly new-onset or worsening heart failure (27.9%), septic embolism (25.1%), and cerebral embolism (18.9%), are highly prevalent and critically dictate patient survival; and (3) identifying independent predictors of survival through multivariable Cox proportional hazards regression analysis established that hospital-acquired IE (HR = 2.247; *p* = 0.010) alongside specific major clinical complications, notably sepsis (HR = 2.746; *p* = 0.001), acute kidney injury (HR = 2.479; *p* = 0.003), cranial embolism (HR = 1.971; *p* = 0.034), and myocardial infarction (HR = 2.200; *p* = 0.032), emerges as the strongest independent determinant of mortality in this contemporary nationwide cohort.

The epidemiological landscape of IE has evolved significantly over the past two decades. Historically dominated by oral streptococci and rheumatic heart disease, contemporary IE is increasingly driven by staphylococci and iatrogenic healthcare exposures [1,2,4]. Our cohort rigorously corroborates this paradigm shift. While hospital-acquired IE accounted for 41.1% of our cases, *S. aureus* was the most frequently identified pathogen (27.9%), with an alarming proportion of these isolates (38.9%) being methicillin-resistant (MRSA). Furthermore, the high prevalence of central venous catheters (25.5%), chronic hemodialysis (24.5%), and recent hospitalizations within the preceding six months (45.2%) underscores the mounting burden of medical interventions as predisposing substrates. These findings align closely with broader international data, such as the EURO-ENDO registry conducted in a multicenter setting between January 2016 and March 2018, which included 3116 adult patients diagnosed with IE according to the ESC 2015 diagnostic criteria. Infective endocarditis was identified as NVE in 1764 (56.6%) patients, PVIE in 939 (30.1%) patients, and device-related (CDRIE) in 308 (9.9%) patients. Infective endocarditis was community-acquired in 2046 (65.66%) patients. The associated microorganisms were *Staphylococcus* in 1085 (44.1%) patients, oral streptococci in 304 (12.3%) patients, *Enterococcus* in 390 (15.8%) patients, and *Streptococcus gallolyticus* in 162 (6.6%) patients. Embolic events occurred in 20.6% of patients. According to ESC guidelines, 2160 (69.3%) patients indicated cardiac surgery, but only 1596 (73.9%) ultimately underwent it [10]. Despite significant advances in diagnostic imaging and surgical techniques, mortality associated with IE remains unacceptably high. In our cohort, 28-day mortality was 11.9%, rising to 26.4% during the follow-up period. In the EURO-ENDO registry, hospital death occurred in 532 (17.1%) patients [10]. Both our study’s findings and the EURO-ENDO registry highlight the urgent need for stringent infection control measures and targeted antimicrobial management in high-risk healthcare settings.

In our study, hospital-acquired infective endocarditis (IE) was significantly more prevalent among non-survivors compared to survivors (59.7% vs. 34.4%, *p* < 0.001). Similarly, Kaplan–Meier survival analysis confirmed that overall survival was markedly worse in patients with hospital-acquired IE than in those with community-acquired IE (log-rank *p* < 0.001). These findings indicate that hospital-acquired IE should not be regarded merely as an epidemiological classification, but rather as a high-risk clinical phenotype associated with adverse outcomes. Several underlying mechanisms contribute to this unfavorable prognosis, including exposure to central venous catheters and invasive devices, chronic hemodialysis, recent hospitalization, and a higher baseline burden of comorbidities [11,12]. Furthermore, healthcare-associated acquisition often increases the likelihood of resistant pathogens, leads to diagnostic delays, and complicates effective source control. Consequently, this vulnerable patient population requires prompt multidisciplinary evaluation and intensive clinical monitoring [11,12].

In patients with suspected IE, current guidelines recommend initiating empirical antimicrobial therapy as soon as possible after blood cultures are taken [1,5,8]. In our study, empirical antibiotics were initiated in the majority of patients (89.8%), with vancomycin (45.7%) and third-generation cephalosporins (36.9%) being the most frequently preferred agents. However, the fact that the appropriateness rate of empirical treatment remained at 51.2% and inappropriate empirical treatment was applied in 40.3% of cases indicates a significant need for optimizing empirical regimens in real-world clinical practice by considering the pathogen spectrum and local resistance patterns. Although traditional recommendations mandate emergency treatment in suspected cases, recent clinical evaluations question the rigidity of this approach in certain subgroups. A recent study investigating the clinical benefit of emergency treatment in hemodynamically stable IE patients did not include patients presenting with sepsis, intensive care unit admission, neutropenia, or a clearly identifiable focus other than IE. In 675 (55%) cases, empirical antimicrobial treatment was initiated immediately after blood culture, and in 555 (45%) cases, treatment was delayed until preliminary blood culture results were available. No significant difference was found between the groups in terms of 30-day mortality and the composite primary endpoint (5% vs. 5%, *p* = 0.894; 28% vs. 24%, *p* = 0.304, respectively) [13]. Although this valuable study in the literature predominantly focused on selected and hemodynamically stable profiles, our study successfully reflects the complex clinical spectrum of infective endocarditis owing to a large real-life cohort including high-risk patients requiring intensive care unit admission. As shown in the COSMO-IE study in Table 9, neither initiating empirical antibiotics nor their initial appropriateness appeared to directly alter overall prognosis, a finding consistent with previous report [13]. Rather, our findings indicate that, while initial empirical choices were made, non-survivors exhibited a lack of clinical response, which subsequently led clinicians to attempt treatment modifications significantly more often. Therefore, the higher frequency of treatment modifications in the non-survivor group (25.8% vs. 12.4%, *p* = 0.001) and the lower rates of favorable clinical response at day 7 in this group reflect refractory clinical worsening and highlight the vital importance of adopting correct and targeted antimicrobial strategies promptly.

Echocardiographic findings in patients with infective endocarditis (IE) serve as the cornerstone of the diagnostic process and play a critical role in patients’ risk stratification and the determination of therapeutic strategies [14,15]. The size, morphology, and localization of vegetation detected via echocardiography are particularly decisive factors in terms of the risk of embolic events and hemodynamic deterioration. Furthermore, early echocardiographic detection of complications, such as valvular destruction, new-onset valvular regurgitation, or perivalvular abscess, is important for timely surgical indications [1,14,15]. In a study involving 779 patients with IE (66.4% male, mean age 65.0 years), symptomatic embolic events occurred in 234 (30%) patients. Multivariate analysis identified highly mobile vegetation as the strongest predictor of embolism risk (*p* < 0.001) [16]. In our study, mobile vegetation (76.6% vs. 53.5%, *p* < 0.001) and moderate-to-severe valvular insufficiency (64.5% vs. 46.2%, *p* < 0.001) were found to be higher in the non-survivor group compared to the survivor group. However, in our multivariable regression models incorporating the composite clinical complications variable, which includes embolic episodes, mobile vegetation did not emerge as an independent predictor of mortality. As methodological considerations dictate, because mobile vegetation is a primary anatomical precursor of systemic and cerebral embolic events, the inclusion of embolic complications within our composite endpoint likely attenuated the independent prognostic variance attributable to vegetation mobility. Consequently, while echocardiographic parameters remain indispensable for assessing local tissue damage and acute embolic risk, their direct independent association with mortality in multivariable models can be statistically influenced by downstream complications.

Regarding the microbiological etiology, our results demonstrated that *Candida* spp. infections were associated with the highest mortality rates among all groups (Figure 1). This observation is consistent with the aggressive clinical course typically documented in fungal endocarditis, which often involves extensive valve destruction and embolic complications [17]. Similarly, *Staphylococcus aureus* endocarditis in our cohort continued to present with a significantly poor prognosis compared to other bacterial pathogens. In contrast, the relatively lower mortality observed in culture-negative endocarditis cases, as highlighted in Figure 1, may reflect a more indolent disease presentation or the successful initiation of empiric antibiotic therapy prior to microbiological diagnosis. These findings reinforce the necessity of early identification and aggressive management strategies for patients presenting with high-risk pathogens such as *Candida* spp. and *S. aureus*, as suggested in recent large-scale registries [10,14].

Multivariate Cox regression analysis revealed that the presence of significant clinical complications (new-onset or worsening heart failure, arrhythmia, sepsis, septic embolism, cerebral embolism, spondylodiscitis, glomerulonephritis, immune complex nephritis, acute kidney injury, splenic abscess, pulmonary embolism, myocardial infarction) independently increased the long-term mortality risk by more than four times (HR = 4.207, 95% CI: 1.556–11.378). This finding is in complete agreement with the literature, emphasizing that organ failures and additional complications play a central role in determining long-term prognosis in IE cases. In a large-scale national cohort study of endocarditis complications obtained using ICD codes, 38.2% of adult IE patients (mean age 60.3 ± 20.1 years, 48.5% female) developed one or more major complications. The most common major complications were cardiovascular complications (22.6%), neurological complications (16.7%), and renal complications (6.4%). Patients with complicated infective endocarditis had a higher risk of death (adjusted RR 1.12, 95% CI 1.11–1.13, *p* <0.001). Complications strongly associated with death were septic shock (RR 1.29, 95% CI 1.27–1.30, *p* < 0.001), cardiogenic shock (RR 1.24, 95% CI 1.20–1.29, *p* < 0.001), disseminated intravascular coagulation (DIC) (RR 1.4, 95% CI 1.35–1.46, *p* < 0.001), and systemic thromboembolism (RR 1.07, 95% CI 1.05–1.08, *p* < 0.001) [17]. In this large-scale national cohort study conducted by Mir et al., it was reported that the risk of mortality increased exponentially in IE patients who developed complications, and the vast majority of observed deaths could be directly attributed to these complications [18].

A study on a prospective case series population was conducted to define the epidemiological, clinical, and microbial characteristics of critically ill patients admitted to the ICU with a definite diagnosis of IE, which included 325 patients (231 [71.08%] in the ward, 94 [28.92%] in the ICU). Following univariate and multivariate logistic regression analyses, the independent predictors of ICU admission were identified as vegetation diameter > 10 mm, abnormal PaO2/FiO2 ratio, and acute heart failure; furthermore, five independent mortality risk factors were identified: SOFA score > 14, absence of surgery, age > 70 years, acute heart failure, and embolic complications [19]. Similarly, in a single-center study conducted by Kazancıoğlu et al., it was emphasized that additional events leading to the worsening of the clinical status, such as heart failure and renal dysfunction, were among the strongest independent predictors of both in-hospital and long-term mortality [20]. The high risk ratio (HR = 4.207) obtained in our study confirms that systemic and cardiac complications occurring in the acute phase significantly threaten not only the short-term survival of patients but also their long-term post-discharge prognosis. In this context, early diagnosis of organ involvement and complications, along with the adoption of aggressive medical/surgical management strategies in the follow-up of IE patients, plays a key role in reducing mortality.

Several limitations of our study should be acknowledged. First, the retrospective, observational design inherently limits our analysis to previously recorded clinical variables. Second, while the COSMO-IE study successfully captures clinical and survival outcomes, detailed data regarding post-discharge functional status and quality of life were not systematically evaluated. Third, given the extensive spectrum of parameters evaluated in our multivariable regression models, analyzing each major complication separately in a single model was initially constrained by statistical overfitting and events-per-variable ratios. Consequently, our analysis utilized a composite clinical complications variable encompassing diverse events such as heart failure, sepsis, embolic episodes, acute kidney injury, and arrhythmias. Importantly, because these major complications developed during the clinical course rather than at baseline, this composite complication variable functions as an intermediate, time-dependent risk factor rather than a pure baseline predictor, which may inherently inflate hazard ratios due to conditional associations. Furthermore, due to limited event numbers within specific early sub-intervals, performing a formal 7-day landmark analysis or a separate 28-day mortality model was statistically unfeasible without compromising statistical power. Although we presented the univariable and multivariable associations of individual major complications to help readers interpret the driving components, the intermediate and time-dependent nature of these events must be considered when interpreting their prognostic impact. Fourth, despite the nationwide, multicenter design involving 27 centers across Turkey, potential center-level variations in diagnostic thresholds, antimicrobial protocols, surgical timing, and Endocarditis Team management may introduce institutional heterogeneity. Although performing a formal statistical adjustment for center-level grouping was precluded by the distribution of cases across participating sites, this real-world representation reflects routine clinical practice under diverse local resistance profiles. In this context, the rate of appropriate empirical antibiotic therapy observed in approximately half of our cases highlights a critical area for clinical improvement, while simultaneously enhancing the generalizability of our findings. Finally, patients with refractory cardiogenic shock or previous out-of-hospital cardiopulmonary resuscitation were excluded to prevent extreme mortality confounding from masking primary risk factors, which naturally limits generalizability to catastrophic or terminal presentations.

## 5. Conclusions

Contemporary infective endocarditis is a highly complex and fatal disease characterized by a prominent healthcare-associated burden and a predominant staphylococcal etiology. The occurrence of major clinical complications and hospital-acquired infections were the primary independent predictors of mortality. These findings emphasize that early identification of high-risk patients, rigorous monitoring for systemic complications, rapid microbiological diagnosis, and optimized therapeutic management, particularly targeting healthcare-associated infections and virulent pathogens, are essential to improving survival and clinical prognosis in infective endocarditis.

## Figures and Tables

**Table 1:** Baseline demographic and clinical characteristics of the study population.

Variable	Total (*n* = 470)
Age, years	58.08 ± 16.81
Sex	
Female	185/470 (39.4)
Male	285/470 (60.6)
Comorbidities	
Hypertension	267/470 (56.8)
Diabetes mellitus	168/470 (35.7)
Atrial fibrillation	65/470 (13.8)
Chronic obstructive pulmonary disease	31/470 (6.6)
Congenital heart disease	19/470 (4.0)
Coronary artery disease	153/470 (32.6)
Congestive heart failure	124/470 (26.4)
Chronic kidney disease	162/470 (34.5)
Rheumatologic disease	46/470 (9.8)
Chronic liver disease	12/470 (2.6)
History of stroke or transient ischemic attack	96/470 (20.4)
Hematologic malignancy	7/470 (1.5)
Solid tumor	26/470 (5.5)
Major trauma within the previous month	1/470 (0.2)
Immunosuppression	34/470 (7.2)

**Table 2:** Clinical classification, acquisition setting, anatomical distribution, and predisposing conditions of infective endocarditis.

Type of Infective Endocarditis	
Native valve endocarditis	309/470 (65.7)
Prosthetic valve endocarditis	115/470 (24.5)
Cardiac implantable device-related endocarditis	31/470 (6.6)
Catheter-related endocarditis	15/470 (3.2)
Acquisition setting	
Community-acquired endocarditis	277/470 (58.9)
Hospital-acquired endocarditis	193/470 (41.1)
Anatomical distribution	
Mitral valve	226/470 (48.1)
Aortic valve	143/470 (30.4)
Tricuspid valve	55/470 (11.7)
Pulmonary valve	2/470 (0.4)
Lead-related endocarditis	20/470 (4.3)
Other	24/470 (5.1)
Predisposing conditions	
Previous infective endocarditis	19/470 (4.0)
Recent dental procedure	68/470 (14.5)
Intravenous drug use	13/470 (2.8)
Cardiac implantable electronic device	40/470 (8.5)
Valvular heart disease	238/470 (50.6)
Prosthetic valve	122/470 (26.0)
Central venous catheter	120/470 (25.5)
Hemodialysis	115/470 (24.5)
Hospitalization within the previous 6 months	212/470 (45.2)

Data are presented as *n*/N (%), with variable-specific denominators reflecting the number of patients with available data. Some categories were not mutually exclusive; therefore, percentages may not sum to 100%.

**Table 3:** Microbiological profile and antimicrobial susceptibility patterns of infective endocarditis.

Variable	Total (*n* = 470)
Causative microorganisms	
*Staphylococcus aureus*	131/470 (27.9)
*Staphylococcus* spp.	45/470 (9.6)
Coagulase-negative staphylococci	57/470 (12.1)
*Streptococcus* spp.	37/470 (7.9)
*Enterococcus* spp.	63/470 (13.4)
Enterobacterales	16/470 (3.4)
Non-fermentative Gram-negative bacilli	2/470 (0.4)
*Candida* spp.	11/470 (2.4)
*Brucella* spp.	3/470 (0.6)
Other microorganisms	22/470 (4.7)
Culture-negative infective endocarditis	74/470 (15.7)
Polymicrobial/other unclassified infection	9/470 (1.9)
Methicillin susceptibility among staphylococcal isolates, *n*/N (%)	
Methicillin-resistant	105/232 (45.3)
Methicillin-susceptible	127/232 (54.7)
Methicillin susceptibility among *S. aureus* isolates, *n*/N (%)	
MRSA	51/131 (38.9)
MSSA	80/131 (61.1)

Data are presented as *n*/N (%), with variable-specific denominators reflecting the number of patients with available data. Abbreviations: MRSA, methicillin-resistant *Staphylococcus aureus*; MSSA, methicillin-susceptible *Staphylococcus aureus*; *n*/N, number of isolates with the specified susceptibility pattern/total number of isolates tested.

**Table 4:** Clinical presentation, echocardiographic findings, and empirical antimicrobial therapy.

Variable	Total (*n* = 470)
Clinical presentation	
Fever	289/470 (61.5)
Dyspnea	211/470 (44.9)
Chest pain	94/470 (20.0)
Syncope	43/470 (9.1)
Arthralgia	103/470 (21.9)
Myalgia	123/470 (26.2)
Echocardiographic findings	
Mobile vegetation	280/470 (59.6)
Paravalvular abscess	30/470 (6.4)
Paravalvular leak	94/470 (20.0)
Flail leaflet	57/470 (12.1)
Prosthetic valve dehiscence	38/470 (8.1)
Moderate-to-severe valvular regurgitation	240/470 (51.1)
Empirical antimicrobial therapy	422/470 (89.8)
Empirical antimicrobial agents	
Third-generation cephalosporin	173/470 (36.9)
Amoxicillin	17/470 (3.6)
Ampicillin	57/470 (12.1)
Gentamicin	82/470 (17.4)
Vancomycin	215/470 (45.7)
Cefazolin	23/470 (4.9)
Daptomycin	26/470 (5.5)
Fosfomycin	4/470 (0.9)
Ertapenem	4/470 (0.9)
Levofloxacin	11/470 (2.3)
Appropriateness of empirical antimicrobial therapy	
Inappropriate	170/422 (40.3)
Appropriate	216/422 (51.2)
Not assessable	36/422 (8.5)

Data are presented as *n*/N (%), with variable-specific denominators reflecting the number of patients with available data. Data on empirical antibiotic therapy were available for 422 of the 470 patients due to missing documentation in 48 cases. Patients could receive more than one empirical antimicrobial agent; therefore, percentages for individual agents and imaging modalities may exceed 100%.

**Table 5:** Complications, treatment modifications, and clinical and microbiological responses.

Variable	Total (*n* = 470)
New-onset or worsening heart failure	131/470 (27.9)
Arrhythmia	58/470 (12.3)
Sepsis	98/470 (20.9)
Septic embolism	118/470 (25.1)
Cerebral embolism	89/470 (18.9)
Spondylodiscitis	23/470 (4.9)
Glomerulonephritis	9/470 (1.9)
Splenic abscess	25/470 (5.3)
Pulmonary embolism	20/470 (4.3)
Immune complex nephritis	15/470 (3.2)
Acute kidney injury	63/470 (13.4)
Myocardial infarction	26/470 (5.5)
Treatment modification	
Culture result-guided treatment modification	224/449 (49.9)
Treatment modification due to inadequate response	73/460 (15.9)
Treatment modification due to adverse effects	45/459 (9.8)
Treatment response	
Clinical response by day 7	311/463 (67.2)
Microbiological response	290/396 (65.7)
Supportive care and nutrition	
Endotracheal intubation	79/470 (16.8)
Total parenteral nutrition	33/470 (7.0)
Treatment	
Surgical treatment	191/470 (40.6)
Outpatient management	119/470 (25.3)
Mortality outcomes	
28-day mortality	56/470 (11.9)
Mortality during follow-up	124/470 (26.4)

Data are presented as *n*/N (%), with variable-specific denominators reflecting available data. Patients may have experienced more than one complication; therefore, percentages do not sum to 100%.

**Table 6:** Comparison of clinical characteristics according to mortality status during follow-up.

Variable	Survivors (*n* = 346)	Non-Survivors (*n* = 124)	*p* Value
Age, years	56.42 ± 16.38	62.73 ± 17.17	**<0.001**
Male sex	212/346 (61.3)	73/124 (58.9)	0.639
Type of infective endocarditis			
Native valve endocarditis	231/346 (66.8)	78/124 (62.9)	0.437
Prosthetic valve endocarditis	81/346 (23.4)	34/124 (27.4)	0.373
Cardiac implantable electronic device-related endocarditis	22/346 (6.4)	9/124 (7.3)	0.729
Catheter-related endocarditis	13/346 (3.8)	2/124 (1.6)	0.244
Hospital-acquired endocarditis	119/346 (34.4)	74/124 (59.7)	**<0.001**
Endocarditis localization			0.532
Mitral valve	163/346 (47.1)	63/124 (50.8)	
Tricuspid valve	44/346 (12.7)	11/124 (8.9)	
Aortic valve	104/346 (30.1)	39/124 (31.5)	
Pulmonary valve	2/346 (0.6)	0/124 (0.0)	
Lead-related endocarditis	13/346 (3.8)	7/124 (5.6)	
Other	20/346 (5.8)	4/124 (3.2)	
Predisposing conditions and healthcare exposures			
History of infective endocarditis	15/346 (4.3)	4/124 (3.2)	0.590
Valvular heart disease	163/346 (47.1)	75/124 (60.5)	**0.011**
Prosthetic valve	87/346 (25.1)	35/124 (28.2)	0.502
Central venous catheter	78/346 (22.5)	42/124 (33.9)	**0.013**
Cardiac implantable electronic device	27/346 (7.8)	13/124 (10.5)	0.359
Congenital heart disease	17/346 (4.9)	2/124 (1.6)	0.109
Intravenous drug use	11/346 (3.2)	2/124 (1.6)	0.529 ᵃ
Recent dental procedure	53/346 (15.3)	15/124 (12.1)	0.382
Hemodialysis	78/346 (22.5)	37/124 (29.8)	0.105
Previous hospitalization	151/345 (43.8)	61/124 (49.2)	0.298
Immunosuppressive treatment within the previous 3 months	22/346 (6.4)	9/124 (7.3)	0.729
Comorbidities			
Coronary artery disease	108/346 (31.2)	45/124 (36.3)	0.301
Heart failure	81/346 (23.4)	43/124 (34.7)	**0.015**
Hypertension	189/346 (54.6)	78/124 (62.9)	0.110
Atrial fibrillation	46/346 (13.3)	19/124 (15.3)	0.575
Chronic kidney disease	117/346 (33.8)	45/124 (36.3)	0.619
Diabetes mellitus	117/346 (33.8)	51/124 (41.1)	0.145
Rheumatologic disease	33/346 (9.5)	13/124 (10.5)	0.761
Chronic liver disease	10/346 (2.9)	2/124 (1.6)	0.740 ᵃ
History of stroke or transient ischemic attack	69/346 (19.9)	27/124 (21.8)	0.664
Chronic obstructive pulmonary disease	22/346 (6.4)	9/124 (7.3)	0.729
Hematologic malignancy	5/346 (1.4)	2/124 (1.6)	1.000 ᵃ
Solid tumor	16/346 (4.6)	10/124 (8.1)	0.150
Immunosuppression	21/346 (6.1)	13/124 (10.5)	0.103

Data are presented as *n*/N (%), with variable-specific denominators reflecting the number of patients with available data. Categorical variables were compared using Pearson’s chi-square test. ᵃ The two-sided Fisher’s exact test was used for hematologic malignancy, intravenous drug use, chronic liver disease, and catheter-related endocarditis because at least one expected cell count was less than 5. A two-sided *p* value < 0.05 was considered statistically significant. Note: Values in bold indicate statistical significance.

**Table 7:** Clinical and echocardiographic findings according to mortality status during follow-up.

Variable	Survivors (*n* = 346)	Non-Survivors (*n* = 124)	*p* Value
Clinical presentation			
Fever	218/346 (63.0)	71/124 (57.3)	0.259
Dyspnea	145/346 (41.9)	66/124 (53.2)	**0.030**
Chest pain	74/346 (21.4)	20/124 (16.1)	0.209
Syncope	31/346 (9.0)	12/124 (9.7)	0.812
Arthralgia	81/346 (23.4)	22/124 (17.7)	0.190
Myalgia	95/346 (27.5)	28/124 (22.6)	0.289
Fever duration, days	10.97 ± 16.16	9.96 ± 14.03	0.639
Echocardiographic findings			
Left ventricular ejection fraction, %	52.21 ± 11.59	50.59 ± 12.11	0.189
Systolic pulmonary artery pressure, mmHg	34.59 ± 13.57	38.34 ± 15.63	**0.025**
Paravalvular abscess	18/346 (5.2)	12/124 (9.7)	0.080
Paravalvular leak	65/346 (18.8)	29/124 (23.4)	0.272
Flail leaflet	42/346 (12.1)	15/124 (12.1)	0.990
Prosthetic valve dehiscence	25/346 (7.2)	13/124 (10.5)	0.253
Moderate-to-severe valvular regurgitation	160/346 (46.2)	80/124 (64.5)	**<0.001**
Hospital stay, days	27.0 [14.0–42.0]	28.0 [12.8–40.3]	0.929
ICU length of stay, days	7.0 [3.0–13.0]	12.0 [5.0–24.0]	**0.002**
Post-discharge follow-up duration, months	13.0 [6.0–20.0]	3.0 [0.6–9.0]	**<0.001**

Data are presented as *n*/N (%), with variable-specific denominators reflecting the number of patients with available data. Percentages were calculated within mortality groups. Between-group comparisons were performed using Pearson’s chi-square test. A two-sided *p*-value < 0.05 was considered statistically significant. Note: Values in bold indicate statistical significance.

**Table 8:** Comparison of laboratory parameters according to mortality status during follow-up.

Variable	Survivors, (*n* = 346)	Non-Survivors, (*n* = 124)	*p* Value
Creatinine, mg/dL	1.19 [0.83–2.89]	1.60 [0.99–4.15]	**0.013**
eGFR, mL/min/1.73 m^2^	63.0 [22.0–91.0]	41.0 [12.5–71.0]	**<0.001**
CRP, mg/L	78.0 [14.0–153.0]	109.0 [48.0–207.3]	**0.001**
Procalcitonin, ng/mL	1.18 [0.21–5.62]	1.70 [0.38–7.76]	0.073
Erythrocyte sedimentation rate, mm/h	43.0 [26.0–70.5]	44.0 [28.2–76.0]	0.570
ALT, U/L	19.0 [11.0–32.8]	20.0 [11.0–35.8]	0.935
AST, U/L	25.0 [18.0–45.8]	32.0 [17.1–54.0]	0.117
ALP, U/L	89.0 [66.0–135.0]	87.0 [70.0–127.5]	0.420
GGT, U/L	39.5 [24.0–80.5]	45.0 [25.5–68.5]	0.544
Total bilirubin, mg/dL	0.61 [0.40–0.98]	0.64 [0.47–1.22]	0.170
BNP, pg/mL	3473.5 [937.5–11,256.5]	7104.5 [562.4–14,185.8]	0.354
White blood cell count, ×10^9^/L	11.72 ± 5.63	13.24 ± 6.50	**0.022 ᵃ**
Platelet count, ×10^9^/L	227.72 ± 120.37	211.25 ± 126.76	0.199
Lymphocyte count, ×10^9^/L	1.51 ± 1.38	1.37 ± 1.06	0.299
Hemoglobin, g/dL	10.77 ± 2.43	10.02 ± 2.12	**0.002 ᵃ**
Albumin, g/dL	3.32 ± 1.00	3.09 ± 0.87	**0.034**

Data are presented as mean ± standard deviation or median [interquartile range], as appropriate. Between-group comparisons were performed using the independent-samples *t*-test or Mann–Whitney U test, according to the distribution of the data. ᵃ When Levene’s test indicated heterogeneity of variances, Welch’s *t*-test was used for white blood cell count and hemoglobin. A two-sided *p*-value < 0.05 was considered statistically significant. Abbreviations: ALP, alkaline phosphatase; ALT, alanine aminotransferase; AST, aspartate aminotransferase; BNP, B-type natriuretic peptide; CRP, C-reactive protein; eGFR, estimated glomerular filtration rate; GGT, gamma-glutamyl transferase. Note: Values in bold indicate statistical significance.

**Table 9:** Antimicrobial management, clinical complications, and treatment strategies according to mortality status during follow-up.

	Survivors (*n* = 346)	Non-Survivors (*n* = 124)	*p* Value
Any empirical antibiotic therapy	305/346 (88.2)	117/124 (94.4)	0.050
Third-generation cephalosporin	127/346 (36.7)	46/124 (37.1)	0.891
Amoxicillin	13/346 (3.8)	4/124 (3.2)	1.000 ᵃ
Ampicillin	39/346 (11.3)	18/124 (14.5)	0.342
Gentamicin	63/346 (18.2)	19/124 (15.3)	0.468
Vancomycin	150/346 (43.4)	65/124 (52.4)	0.082
Cefazolin	17/346 (4.9)	6/124 (4.8)	0.974
Daptomycin	17/346 (4.9)	9/124 (7.3)	0.327
Fosfomycin	1/346 (0.3)	3/124 (2.4)	0.058 ᵃ
Ertapenem	3/346 (0.9)	1/124 (0.8)	1.000 ᵃ
Levofloxacin	7/346 (2.0)	4/124 (3.2)	0.492 ᵃ
Appropriate empirical therapy	152/286 (53.1)	59/100 (59.0)	0.312
Treatment modification according to culture results	165/331 (49.8)	59/118 (50.0)	0.978
Treatment modification due to lack of response	42/340 (12.4)	31/120 (25.8)	**0.001**
Treatment modification due to adverse effects	37/339 (10.9)	8/120 (6.7)	0.179
Clinical response on day 7	255/341 (74.8)	56/122 (45.9)	**<0.001**
Microbiological response	226/342 (66.1)	64/121 (52.9)	**0.010**
Clinical complications			
New-onset or worsening heart failure	84/346 (24.3)	47/124 (37.9)	**0.004**
Arrhythmia	28/346 (8.1)	30/124 (24.2)	**<0.001**
Sepsis	41/346 (11.8)	57/124 (46.0)	**<0.001**
Septic embolism	53/346 (15.3)	65/124 (52.4)	**<0.001**
Cerebral embolism	42/346 (12.1)	47/124 (37.9)	**<0.001**
Spondylodiscitis	12/346 (3.5)	11/124 (8.9)	**0.017**
Glomerulonephritis	5/346 (1.4)	4/124 (3.2)	0.252 ᵃ
Immune complex nephritis	5/346 (1.4)	10/124 (8.1)	**0.001 ᵃ**
Acute kidney injury	20/346 (5.8)	43/124 (34.7)	**<0.001**
Splenic abscess	11/346 (3.2)	14/124 (11.3)	**0.001**
Pulmonary embolism	7/346 (2.0)	13/124 (10.5)	**<0.001**
Myocardial infarction	11/346 (3.2)	15/124 (12.1)	**<0.001**
Treatment strategies			
Surgical treatment	128/346 (37.0)	63/124 (50.8)	**0.007**
Outpatient treatment selected	107/346 (30.9)	12/124 (9.7)	**<0.001**

Data are presented as *n*/N (%), with variable-specific denominators reflecting the number of patients with available data. Pearson’s chi-square test was used unless otherwise indicated. ᵃ Fisher’s exact test (two-sided) was used for amoxicillin, fosfomycin, ertapenem, levofloxacin, glomerulonephritis, and immune complex nephritis. Percentages for appropriate empirical therapy were calculated among patients who received empirical antimicrobial therapy and for whom treatment appropriateness was assessable. A two-sided *p*-value < 0.05 was considered statistically significant. Note: Values in bold indicate statistical significance.

**Table 10:** Univariable and multivariable Cox proportional hazards regression analysis for factors associated with mortality.

Variables	Univariate Cox Regression			Multivariate Cox Regression		
	HR	95% CI	*p* Value	HR	95% CI	*p* Value
Age, per 1-year increase	1.027	1.011–1.043	**0.001**	1.021	1.000–1.043	0.053
Hospital-acquired IE ^a^	2.101	1.312–3.367	**0.002**	2.247	1.216–4.167	**0.010**
Valvular heart disease	1.590	0.983–2.570	0.059	-	-	-
Central venous catheter	1.461	0.887–2.408	0.136	-	-	-
Chronic heart failure	1.559	0.961–2.529	0.072	-	-	-
Dyspnea	1.506	0.945–2.400	0.085	-	-	-
Mobile vegetation	2.005	1.161–3.461	**0.013**	1.336	0.617–2.892	0.462
Paravalvular abscess	1.678	0.769–3.665	0.194	-	-	-
Valvular regurgitation	1.583	0.980–2.557	0.061	-	-	-
eGFR	0.989	0.983–0.995	**0.001**	1.002	0.992–1.011	0.747
CRP	1.002	1.000–1.004	0.097	-	-	-
Procalcitonin	1.004	0.988–1.020	0.653	-	-	-
WBC	1.035	0.998–1.073	0.063	-	-	-
Hemoglobin	0.842	0.747–0.950	**0.005**	0.911	0.765–1.084	0.292
Albumin	0.934	0.691–1.264	0.659	-	-	-
Systolic pulmonary artery pressure	1.019	1.004–1.035	**0.015**	1.008	0.990–1.026	0.374
ICU length of stay	1.019	1.010–1.028	**<0.001**	1.011	0.998–1.024	0.098
Any empirical antibiotic therapy	1.522	0.659–3.514	0.325	-	-	-
Vancomycin	1.077	0.669–1.732	0.761	-	-	-
Fosfomycin	9.161	2.193–38.269	**0.002**	4.084	0.846–19.711	0.080
Treatment modification due to lack of response	2.593	1.535–4.381	**0.001**	1.720	0.829–3.568	0.145
Clinical response at day 7	0.397	0.247–0.637	**<0.001**	0.866	0.422–1.778	0.694
Microbiological response	0.529	0.328–0.853	**0.009**	0.571	0.299–1.089	0.089
Clinical complications	6.649	3.175–13.925	**<0.001**	4.207	1.556–11.378	**0.005**
Surgical treatment	1.392	0.873–2.219	0.165	-	-	-
Outpatient treatment	0.476	0.297–0.762	**0.002**	0.626	0.283–1.384	0.247

^a^ Community-acquired IE was used as the reference category. Clinical complications were defined as the occurrence of at least one of the following during the index hospitalization: new-onset or worsening heart failure, arrhythmia, sepsis, septic embolism, cerebral embolism, spondylodiscitis, immune complex nephritis, acute kidney injury, splenic abscess, pulmonary embolism, or myocardial infarction. Clinical complications were analyzed as a composite binary variable indicating the presence of at least one prespecified in-hospital complication. Note: Values in bold indicate statistical significance.

## Data Availability

The datasets presented in this study are not publicly available due to multi-center confidentiality and ethical restrictions. The data presented in this study are available on request from the corresponding author.

## Supplementary Materials

The following supporting information can be downloaded at: https://www.mdpi.com/article/10.3390/jcm15197760/s1, Supplementary Table S1. Univariable and multivariable Cox proportional hazards regression analyses evaluating the prognostic impact of individual clinical complications on mortality.

## Abbreviations

The following abbreviations are used in this manuscript:

ALT	Alanine aminotransferase
AMI	Acute myocardial infarction
AST	Aspartate aminotransferase
BNP	B-type natriuretic peptide
CDRIE	Cardiac device-related infective endocarditis
CI	Confidence interval
CIED	Cardiac implantable electronic device
CRP	C-reactive protein
CT	Computed tomography
DIC	Disseminated intravascular coagulation
eGFR	Estimated glomerular filtration rate
ESC	European Society of Cardiology
GDMT	Guideline-directed medical therapy
GGT	Gamma-glutamyl transferase
HR	Hazard ratio
ICU	Intensive care unit
IE	Infective endocarditis
IQR	Interquartile range
MRI	Magnetic resonance imaging
MRSA	Methicillin-resistant *Staphylococcus aureus*
MSSA	Methicillin-susceptible *Staphylococcus aureus*
NVE	Native valve endocarditis
PVIE	Prosthetic valve infective endocarditis
PVL	Paravalvular leak
RR	Risk ratio
SD	Standard deviation
SOFA	Sequential Organ Failure Assessment
TEE	Transesophageal echocardiography
TTE	Transthoracic echocardiography
WBC	White blood cell
