# Supplementary material for: Contemporary Clinical Spectrum and Factors Associated with Mortality in Infective Endocarditis: A Multicenter Cohort Study (COSMO-IE)
Source: J Clin Med. 2026 Oct 8;15(19):7760. doi: 10.3390/jcm15197760 (PMC13666091; doi:10.3390/jcm15197760)
Supplement: Supplementary file 1 [file jcm-15-07760-s001.zip › jcm-4532030-supplementary.pdf]

Supplementary Table S1. Univariable and multivariable Cox proportional hazards regression analyses evaluating the prognostic impact of individual clinical complications on mortality

Variable	Univariate HR (95% CI)	p-value	Multivariate HR (95% CI)	p-value
New-onset or worsening heart failure	1.737 (1.065-2.831)	0.027	1.223 (0.718-2.082)	0.458
Arrhythmia	3.115 (1.821-5.330)	<0.001	1.783 (0.993-3.198)	0.053
Sepsis	5.305 (3.287-8.561)	<0.001	2.746 (1.523-4.953)	**0.001**
Septic Embolism	3.061 (1.917-4.889)	<0.001	1.091 (0.614-1.939)	0.766
Cerebral Embolism	2.984 (1.827-4.874)	<0.001	1.971 (1.051-3.694)	**0.034**
Spondylodiscitis	1.696 (0.731-3.938)	0.219	1.100 (0.456-2.656)	0.832
Glomerulonephritis	1.886 (0.461-7.716)	0.377	0.800 (0.161-3.979)	0.785
Immune Complex Nephritis	2.845 (1.231-6.577)	0.014	2.207 (0.825-5.906)	0.115
Acute Kidney Injury	5.457 (3.371-8.836)	<0.001	2.479 (1.370-4.485)	**0.003**
Splenic Abscess	2.970 (1.471-5.994)	0.002	2.191 (0.998-4.813)	0.051
Pulmonary Embolism	2.623 (1.133-6.072)	0.024	1.299 (0.493-3.421)	0.596
Myocardial Infarction	4.488 (2.394-8.413)	0.032	2.200 (1.072-4.514)	**0.032**
